# Pulmonary delivery of excipient-free tobramycin DPIs for the treatment of *Pseudomonas aeruginosa* lung infection with CF

**DOI:** 10.3389/fphar.2025.1528905

**Published:** 2025-06-17

**Authors:** Song Cheng, Haozhou Huang, Zhihao Zhang, Mateng Chen, Yulong Zhang, Mengxing Lin, Gang Yang, Lynda Thubelihle Kanye, Qingzhen Zhang, Ning Xue, Kaiqi Shi, Bin Dong, Hanhan Li

**Affiliations:** ^1^ School of Engineering, China Pharmaceutical University, Nanjing, China; ^2^ Eastern Institute for Advanced Study, Eastern Institute of Technology, Ningbo, China; ^3^ Courant Institute of Mathematical Sciences, New York University, New York, NY, United States; ^4^ National Heart and Lung Institute, Faculty of Medicine, Imperial College London, London, United Kingdom; ^5^ Suzhou Inhal Pharma Co., Ltd, Suzhou, Jiangsu, China; ^6^ Department of Computer Science, University of Nottingham Ningbo China, Ningbo, Zhejiang, China; ^7^ Engineering Research Center for Smart Pharmaceutical Manufacturing Technologies, Ministry of Education, China Pharmaceutical University, Nanjing, China

**Keywords:** dry powder inhalations (DPIs), spray freeze-drying (SFD), tobramycin, pulmonary drug delivery, *Pseudomonas aeruginosa* infection

## Abstract

*Pseudomonas aeruginosa* infection has become a widespread problem in patients with cystic fibrosis (CF). A safe and effective manufacturing method is required to produce antibiotic dry powder inhalations (DPIs) which can be effectively delivered to treat lung infections. In this study, an excipient-free tobramycin inhalable powder was prepared using spray freeze-drying (SFD) method. The mass median aerodynamic diameters (MMAD) of optimized inhalable powder prepared by SFD was 1.30 µm, and the fine particle fractions (FPF) reached 83.31%. In both *in vitro* and *in vivo* safety and activity studies, the inhalable powder showed excellent safety performance at both animal and cellular levels, with a minimum inhibitory concentration (MIC) of 0.5 μg/mL. Compared with intravenous injection, inhalation of excipient-free tobramycin inhalable powder had a better effect in the infected mouse model because of its amorphous state. This study demonstrates that excipient-free tobramycin inhalable powder with good delivery and deposition performance can be successfully obtained using the SFD method. Inhalation of excipient-free tobramycin inhalable powder has the potential to be a promising strategy for treating pulmonary infections caused by *P. aeruginosa* in patients with CF.

## Graphical Abstract

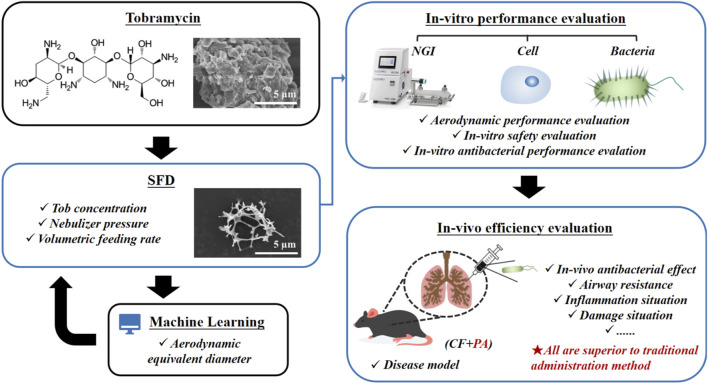

## 1 Introduction

Pulmonary infection has emerged as a prevalent global health concern and societal burden owing to the characteristics of difficult treatment, long disease course, and high mortality rate (Guo et al., 2021; Habibi et al., 2020). Public data from the World Health Organization (WHO) indicate that chronic obstructive pneumonia and lower respiratory tract infections caused by lung infections have become the third and fourth leading causes of death globally, with high mortality rates in infants, young children, and the elderly (Chen et al., 2023; Tesfaw et al., 2021). In 2022, these two diseases caused approximately 8.7 million deaths worldwide (Dead Kicking, 2023). In addition, the escalating number of pandemic coronavirus disease 2019 (COVID-19) infections imposed negative influences on the control of lung diseases, and the suppression of lung diseases became more challenging (Husain-Syed et al., 2021).

The main manifestations of serious lung diseases such as cystic fibrosis (CF) are recurrent bronchial infections and airway obstruction (Harwood et al., 2021). *Pseudomonas aeruginosa* is one of the most common pathogens causing infections. The use of antibiotics for systemic treatment can effectively reduce *P. aeruginosa* infections. In clinical medication, the usual strategies for treating CF rely on oral administration or intravenous (IV) antibiotic combination therapy. However, because of the limited distribution in the lung, these systemic administration methods not only affect drug efficacy but also increase side effects such as bacterial resistance and drug toxicity (Xuan et al., 2023). Furthermore, repeated injections of drugs can cause serious adverse reactions, including nephrotoxicity and ototoxicity (Ren and Fan, 2023). Pulmonary drug delivery is a non-invasive alternative route to treat pulmonary fibrosis caused by *P. aeruginosa* infection by delivering drugs directly to the lungs, resulting in a high local drug concentration at the infection site (Mukker et al., 2015). Therefore, pulmonary drug delivery can minimize systemic exposure and improve drug treatment efficacy, while reducing the risk to patients of drug resistance and development (Chang and Chan, 2021).

Tobramycin is an aminoglycoside antibiotic with advantages such as high water solubility, a wide antibacterial spectrum, definite therapeutic effect, stable properties, and low production cost (Rosalia et al., 2022; Zoratto et al., 2021). PathoGenesis Corporation developed and marketed tobramycin solution spray (trade name: TOBI) in 1998 for *Pseudomonas* pneumonia in cystic fibrosis patients (Rose and Neale, 2010). However, owing to the need for pressure-resistant containers, valve systems, and special production equipment for aerosols, their high cost and difficulty in carrying have raised the threshold for patient use, and they have not been widely popularized. In contrast to metered dose inhalations (MDIs) and other liquid formulations, DPIs exhibit better stability, ease of operation, convenience, and better treatment compliance in patients (Capecelatro et al., 2022). In 2013, tobramycin dry powder inhalations were approved by the FDA for use in patients aged ≥6 years. However, the preparation of DPIs products is often technically challenging. In order to make active ingredients deposit in the lungs and play a local role, the particle size of dry powder must be 0.5–5 μm (Carvalho et al., 2014). Micro-crushing technology is typically used in the industry to produce DPIs. However, this method has many defects, including higher energy consumption, lower efficiency, and wider distribution of product particle size. Spray freeze-drying (SFD) technology (Farinha et al., 2023) offers a solution to the drawbacks of traditional spray drying, which can potentially compromise the efficacy of the drug’s active ingredients owing to high-temperature evaporation during the drying process (Henriques et al., 2022). Additionally, it addresses issues associated with freeze-drying, such as the formation of large-diameter particles with irregular flakes and uneven particle size distribution (Liu et al., 2022). A thermal-inkjet SFD was used by Muennamoon et al. to create excipient-free salbutamol formulations suitable for inhalation (Mueannoom, 2012). Because of the low particle density, a considerable number of particles possessed sufficiently small aerodynamic diameters for pulmonary application, of which FPF approached those of the available commercial products. Lucas D. et al. designed highly porous amorphous celecoxib DPIs by SFD, which were free-flowing, highly spherical (circularity≥ 0.96), accelerating drug absorption *in-vivo* and remaining stable during 6 months of storage (Lucas, 2022).

Many approaches have been applied to improve drug dissolution and bioavailability, including the use of surfactants and the preparation of circular solid dispersions and liposomes. However, the use of excipients, surfactants, or carriers may cause various problems, including low drug-loading capacity and poor stability, as well as unpredictable toxic or adverse reactions such as allergies or other irritation to the respiratory system (Wang, 2023). In contrast, a pure drug preparation can enable patients to obtain sufficient medication at lower doses without additional excipient/carrier to occupy the dosage. Simultaneously, the preparation of pure drug inhalants has streamlined manufacturing procedures, resulting in reduced manufacturing costs and increased operational efficiency, aligning with the principle of green chemistry. The streamlined manufacturing process has led to a reduction in waste generation, diminishing the need for waste disposal and management, which aids in lowering the overall carbon footprint throughout the lifecycle of the inhalant and bolstering the sustainability of the preparation process (Wang, 2020). Therefore, the development of excipient-free antibiotic DPIs suitable for inhalation is crucial. However, notwithstanding the fact that several studies have developed excipient-free tobramycin dry powder inhalants through spray drying or enhanced the stability of their properties by co-spraying with other antibiotics such as colistin, the preparation methods for these formulations either remain quite complex or their aerodynamic parameters still do not reach the expected values. Furthermore, the widespread use of organic reagents in these processes, coupled with the absence of solvent residue detection, has brought about uncertainties concerning both patient physical safety and environmental safety, without providing sufficient assurances (Pilcer et al., 2009; Pathak et al., 2022). If scaled up for production, this problem would become even more pronounced. Solvent recovery and disposal can be complex and costly processes.

To the best of our knowledge, there is no research on the preparation of tobramycin DPIs microparticles without subsidiary material by spray freeze-drying to achieve respirable size for lung delivery. Hence, this study aimed to develop excipient-free tobramycin powders with lower density, improved physical stability, and better aerodynamic performance for pulmonary drug delivery. Tobramycin DPIs were prepared under different operating parameters. The physicochemical properties of the DPIs, including particle size distribution, crystallinity, density, fluidity, and aerosol properties, were systematically evaluated to optimize the manufacturing process. Moreover, an innovative pneumonia model of pulmonary fibrosis in mice infected with *P. aeruginosa* was constructed to investigate the *in vivo* safety, pharmacodynamics, and cytotoxicity.

## 2 Materials and methods

### 2.1 Materials

Tobramycin (C_18_H_37_N_5_O_9,_ molecular weight 467.51 g/mol) was purchased from Hubei Widely Chemical Reagent Co. Ltd (China). Ethanol (HPLC grade, GENERAL-REAGENT^®^) was obtained from Shanghai Titan Scientific Co. Ltd. (China). All other reagents used were of analytical grade. Enzyme-linked immunosorbent assay (ELISA) kits for IL-4, IL-6, and TNF-α were purchased from Hangzhou MultiSciences Biotech Co. Ltd. (China).

### 2.2 Preparation of DPIs by SFD

The operational parameters of the DPIs prepared by SFD are listed in Supplementary Table S1. Different amounts of tobramycin were dissolved in 50 mL of ultrapure water to obtain aqueous solutions of different concentrations (*C*_*tob*_ = 5, 15, 25, 35, and 45 mg/mL). The tobramycin aqueous solution was then transported with a hose (inner diameter = 5 mm) and sprayed into 1 L of liquid nitrogen using a laboratory spray dryer (Shanghai Ya Cheng Co. Ltd., YC-01) at different atomization pressures (*P* = 0.1, 0.15, 0.2, 0.25, and 0.3 MPa) and volumetric feeding rates (*Q* = 18, 21, 24, 27, and 30 mL/min). The sprayed product was then transferred to a lyophilizer (Foring Technology Development Co. Ltd., LGJ-22C) for freeze-drying.

### 2.3 Size and morphology analyses of microparticles

The particle size distribution (PSD) was measured using laser diffraction (DLS) (HELOS/RODOS, SYMPATEC, Germany). Approximately 10 mg of the sample was loaded in a glass dispersion tube and dispersed at a pressure drop of 3.5 bar. An R5 lens with a measuring range of 0.45–875 µm was used for measurement. The values of D_10_, D_50,_ and D_90_ for the volumetric diameter were recorded. Span represents the width of the sample size distribution and was calculated with D_10_, D_50,_ and D_90_ using Equation 1 (Chua et al., 2023):

$$Span=\frac{D_{90}-D_{10}}{D_{50}}$$ (1)

Additionally, for porous or hollow particles, the aerodynamic diameter (*D*_*a*_) can be calculated using the particle tapped density ( $\rho_{t}$ ) and volume median diameter (*VMD*) of the particles using Equation 2 (Alhajj et al., 2021):

$$D_{a}=VMD\sqrt{\rho_{t}}$$ (2)

Scanning electron microscopy (SEM, Hitachi S-4800, Hitachi High-Technologies Crop, Tokyo, Japan) was used to investigate the morphology of the samples. The samples were dispersed on conductive adhesive and imaged in the sample compartment at 5.0 kV for samples.

### 2.4 Flowability study

As an important factor for evaluating inhalation performance, the flowability of particles was investigated via the angle of repose test, in line with the standard protocol specified in the United States Pharmacopoeia (USP). A certain mass of powder often forms a cone when it is affected only by gravity. The steepest angle of descent relative to the horizontal plane is defined as the angle of repose (*θ*). The angle of repose was lower when the sample was non-cohesive and had good flowability. The angle of repose can be calculated using Equation 3 (Ghoroi et al., 2013):

$$\theta =\arctan\;\frac{2H}{D}$$ (3)

where H and D are the powder stacking height and circular stacking diameter, respectively.

In addition, many studies have used the Carr index for characterization and evaluation (Duhan et al., 2021). It is generally believed that the smaller the Carr index, the smaller the compressibility of the powder and the better its fluidity. The Carr index (CI) was calculated using Equation 4:

$$CI\;\left(\%\right)=\frac{\rho_{t}-\rho_{b}}{\rho_{t}}\times 100\%$$ (4)

where ρ_b_ and ρ_t_ are the bulk and tap densities of the sample, respectively.

### 2.5 Powder X-ray diffraction (PXRD)

The crystalline structures of the samples were tested using X-ray powder diffraction (SmartLab-SE, RIGAKU, Japan). Approximately 4 mg of each sample was tightly spread on a sample plate and subjected to Cu radiation at ambient temperature at a current of 15 mA and a voltage of 30 kV. The scanning range of the diffraction angle (2θ) was 5°≤2θ ≤ 60°.

### 2.6 Thermogravimetric analysis (TGA)

Thermogravimetric analysis (TGA) was conducted to analyze the thermal stability of the DPIs samples and the relationship between the sample mass and temperature. Approximately, 3 mg of each sample was weighed and placed in an alumina crucible. The entire system was heated from 30 to 500°C at 10°C/min with a 20 mL/min nitrogen flow in a TGA device (TG 209 F3 Tarsus, NETZSCH, Germany).

### 2.7 Fourier translation infrared spectroscopy (FTIR)

The infrared spectral chemical structures of the samples were analyzed using a Fourier transform infrared spectrometer (Nicolet iS5, Thermo Fisher China Technology, Shanghai, China). The test sample was placed on the “Attenuated Total Reflection” (ATR) detection component of the instrument and the probe was fixed. The scanning frequency was set at 32.

### 2.8 Differential scanning calorimetry (DSC)

The thermal response profiles were obtained using differential scanning calorimetry (DSC) (DSC 214 Polyma, NETZSCH, Germany). The sample was weighed and placed in an aluminum crucible. The entire system was heated from 30°C to 280°C at 10 °C/min in a DSC device under a sustained 50 mL/min nitrogen purge.

### 2.9 *In-Vitro* aerosol performance

According to the British Pharmacopoeia 2017, a next-generation impactor (Logan Instruments, New Jersey, USA) and the DPI inhaler (Carent, China) were used to evaluate the aerosol performance of the DPIs particles. The dispersion rate and duration were 60 L/min and 4 s, respectively. The weight method was used to determine the number of particles deposited at each stage by measuring the weight difference of the glass filter after particle deposition. Emission fraction (EF) is defined as the mass of powder leaving the inhaler relative to the total dose. Fine Particle Fraction (FPF) is defined as the recovered dose, which is the total mass of powders smaller than 5.0 µm. The mass median aerodynamic diameter (MMAD) is defined as the aerodynamic diameter, defining the aerodynamic diameter at which 50% of the particles collected from the first stage to the micropore collector have a larger mass and 50% are smaller. The MMAD and geometric standard deviation (GSD) were calculated by linear fitting of the cumulative mass and aerodynamic cut-off diameter on a logarithmic scale. The aforementioned *in vitro* aerodynamic parameters were collected for each sample and repeated six times.

### 2.10 Cytotoxicity

The human alveolar basal epithelial A549 cell line (Wuxi Xinrun Biotechnology Co., Ltd.) was selected, and the cytotoxicity of the prepared product was evaluated using the MTT (3-(4,5-dimethyl-2-thiazolyl)-2,5-diphenyl-2H-tetrazolium bromide) method. Cell viability was determined by the reduction of MTT to formazan by mitochondria. The cells were cultured in RPMI 1640 medium supplemented with 10% Fetal Bovine Serum (FBS) in 5% CO_2_ atmosphere, and the growth medium was changed every 2–3 days until the cells reached 100% confluence. Cell dissociation and harvesting was performed using trypsin. MTT assay was performed according to the manufacturer’s instructions. Briefly, 190 µL culture medium containing 5,000 cells was inoculated into each well of an experimental 96-well plate, and then incubated for 12 h to achieve 90%–100% confluence. Subsequently, 10 µL of testing products which were obtained by dissolving DPIs sample in PBS with the sample concentrations of 1, 0.5, 10^−1^, 10^−2^, 10^−3^, and 10^−4^ mg/mL were added to the corresponding wells and incubated. After 24-h incubation at 37°C and 5% CO_2_ atmosphere, each well was treated with 10 µL of MTT (5 mg/mL) solution and further incubated for 4 h. After incubation, the culture medium was removed from the wells, and 150 µL of the termination solution, DMSO, was added to each well. After a final incubation period of 30 min, the absorbance of each well was measured at 490 nm. The cell viability (%) was calculated using Equation 5 (Adhikari et al., 2022):

$$Cell\;viability\;\left(\%\right)=\frac{Mean\;of\;Absorbance:value\;of\;treatment\;group}{Mean\;of\;Absorbance:value\;of\;control}\times 100\%$$ (5)

### 2.11 *In-vitro* antibacterial study

The antibacterial activity of the product prepared using the SFD methods against *P. aeruginosa* was evaluated by measuring the minimum inhibitory concentration (MIC) (Arauzo et al., 2021). The growing bacteria were diluted in phosphate-buffered saline (PBS) to a final concentration of approximately 10^7^ CFU/mL. They were then inoculated into the culture media prepared with tobramycin DPIs samples at different concentrations (0, 0.0625, 0.125, 0.250, 0.500, 1, 2, 4, 8, 16, 32, 64, 128, and 256 μg/mL). The bacteria were incubated on a shaking table at 37°C and 150 rpm for 24 h, and the optical density of the pathogenic bacteria in contact with the powder was measured at 600 nm (OD_600_) using a microplate reader (Spectramax@i3x, Molecular Device, United States) to detect bacterial growth.

### 2.12 *In-vivo* safety study

C57BL/6 mice (Hangzhou Ziyuan Laboratory Animal Technology Co., Ltd.), aged 6–8 weeks were used in this study. They were placed and fed under a 12-hour light/12-hour dark cycle. All animal and cell experiments were performed in accordance with the guidelines of the Pharmaceutical Animal Experimental Center of China Pharmaceutical University with the approval number SYXK(SU)-2023–0019.

Mice were divided into healthy groups (six mice), and treated group (six mice), which received tobramycin powders prepared by SFD method 30 mg/kg. The survival rates of each group of mice as well as physical examinations of the skin, eyes, respiratory system, and behavior were observed and recorded every day. The mice were enthanized after a 14-day therapy period. During necropsy, the viscera and body were carefully and thoroughly examined, dissected, gathered, and weighed. In gross necropsies of mice, we meticulously analyzed the body exterior, orifices, and abdominal and thoracic cavities. Dehydrated hearts, livers, spleens, lungs, and kidneys were fixed with 4% paraformaldehyde fixative, embedded in paraffin, sectioned using a microtome at a thickness of 5 μm, mounted on glass slides, stained with hematoxylin and eosin, and prepared for histopathological analysis under a light microscope.

### 2.13 Disease model and dosing

Animals were randomly grouped into five: NC (PBS-treated, negative control, n = 12); BLM (Bleomycin-treated, shortened to BLM-treated, n = 12); BLM + PA (BLM-and-*P. aeruginosa*-treated, n = 12); ITP (BLM-and-*P. aeruginosa*-treated and inhaled sample A4, n = 12); and IV (BLM-and-*P. aeruginosa*-treated and intravenous injection administration of tobramycin, n = 12).

Specifically, BLM, BLM + PA, ITP, and IV groups of animals were treated with a single intratracheal injection of bleomycin (3 mg/kg; Shanghai Yuanye Biotechnology Co., Ltd.) to induce pulmonary fibrosis (Kang et al., 2022). The NC group received an equal amount of PBS. Seventeen days after BLM injection, a bacterial suspension of 50 μL 1 × 10^9^ CFU/mL *P. aeruginosa* was injected into the BLM + PA, ITP, and IV groups. After 1 day of inoculation with PA, group ITP was treated using a dry powder insufflator (DP-M, TOW Intelligent Technology Co., Ltd, China) for intratracheal administration of DPIs sample A4 prepared by SFD method for three consecutive days, and the dosage of administration was 5 mg/kg/dose twice daily. Group IV was administered intravenously for three consecutive days at a dose of 5 mg/kg/dose twice a day. All mice were weighed daily for 25 days. The dosing amount for each group was referenced from the dosing amount of tobramycin in traditional dosage forms used in clinical applications, and was determined according to the conversion of body surface area between humans and experimental animals.

### 2.14 *In-vivo* pharmacodynamics studies

#### 2.14.1 *In-vivo* imaging of the infection

The model bacteria in this study were *P. aeruginosa* with a bioluminescent allowing for visual and quantitative assessment of infection and treatment efficacy in model animals. At a specific time point after administration, the mice were anesthetized by inhaling isoflurane and the intensity was detected using a small animal living imaging system (IVIS Lumina III, PerkinElmer Company, United States). In addition, each animal in each group was sacrificed after the experiment, so that the main organs could be harvested to detect the intensity *ex vivo*. Living Image 4.3 software (PerkinElmer, Waltham, MA, United States) was used for luminescence quantification (radiance [p/sec/cm^2^/sr]). Bioluminescence settings and a luminous exposure time of 60 s were set.

#### 2.14.2 Morphometric analysis of lungs

Lungs and other tissues were removed from all mice and cleaned with PBS to eliminate surface stains before being stored for more than 48 h in a 4% formaldehyde fixation solution for histopathological investigation. Dehydrated tissues were first fixed with 4% paraformaldehyde fixative, embedded in paraffin, sectioned using a microtome at a thickness of 5 μm, mounted on glass slides, stained with hematoxylin and eosin, and prepared for histopathological analysis under a light microscope.

The lung index was used as a reference indicator to evaluate the degree of pulmonary edema. The data were weighed and recorded before taking the material, and the wet lung weights were measured immediately after removing the lung tissue. The lung indices were calculated using Equation 6:

$$Lung\;index=100\times Lung\;wet\;weight\;\left(g\right)/Body\;weight\;\left(g\right)\times 100\%$$ (6)

#### 2.14.3 Enumeration of bacterial load of lungs

After 96 h of lung infection, the lungs of mice were isolated in the biosafety cabinet. The lungs were cut into small pieces, added to 1 mL sterile PBS, and thoroughly homogenized in an ice bath until they were evenly dispersed. Subsequently, the homogenate was filtered to remove the solid substances, and 2 mL of sterile pre-cooled PBS (4 °C) was added for future use. The diluted homogenate (200 μL) was inoculated into 9,800 μL of LB liquid medium (streptomycin, 2000 mg/mL). After 24 h of cultivation in a constant-temperature shaker at 37 °C and 120 rpm, 1 mL of the cultured bacterial solution was centrifuged at 500 G for 10 min to remove the culture medium. The bacterial precipitate was resuspended in 1 mL of sterile PBS, and the samples of each group were measured at 600 nm (OD_600_) to detect bacterial growth. The bacteria were diluted to 10^−7^ CFU/mL which made the differences in bacterial growth among different groups clear, and then inoculated with different samples in peptone agar medium.

#### 2.14.4 Whole body plethysmography test penh value

After treatment, the lung function of all groups of mice was determined by whole-body plethysmography (WBP) (TOW Intelligent Technology Co., Ltd, China Shanghai). The operation of the WBP relies on core pneumotachographs to estimate the results of respiratory functions in experimental animals by measuring the time shift flow between the chest and nasal cavity. Indicators for evaluating lung function include the respiratory rate, duration, volume, and flow rate. This study evaluated the therapeutic effect of drugs based on the size of airway resistance in mice, with a Penh value that considers four breathing coefficients: peak expiratory flow of breath, peak inspiratory flow of breath, time of the expiratory portion of breath, and time required to exhale 65% of the breath volume (Kang et al., 2022). The Penh value was calculated using the WBP machine algorithms based on these four values.

#### 2.14.5 Enzyme-linked immunosorbent assay (ELISA)

The levels of proinflammatory and anti-inflammatory cytokines were measured as an index of inflammation in the pulmonary airways. The mice were anesthetized with 2% isoflurane on day 25. To obtain bronchoalveolar lavage fluid (BALF), the thoracic cavities were opened, the trachea was cannulated, and the lungs were lavaged three times with 400 µL sterile PBS solution. All lavages were collected and centrifuged at 4,000 rpm for 10 min. According to the manufacturer’s instructions, the concentrations of various cytokines, including IL-4, IL-6, and TNF-α, in the BALF were measured using a commercially available Cytokine ELISA kit (MultiSciences Biotech, Hangzhou, China). The concentrations of each cytokine in each BAL sample were calculated in relation to the standard curves, which were created using serially diluted recombinant standard cytokines.

## 3 Results and discussions

### 3.1 Characterization of tobramycin microparticles

**FIGURE 1:**
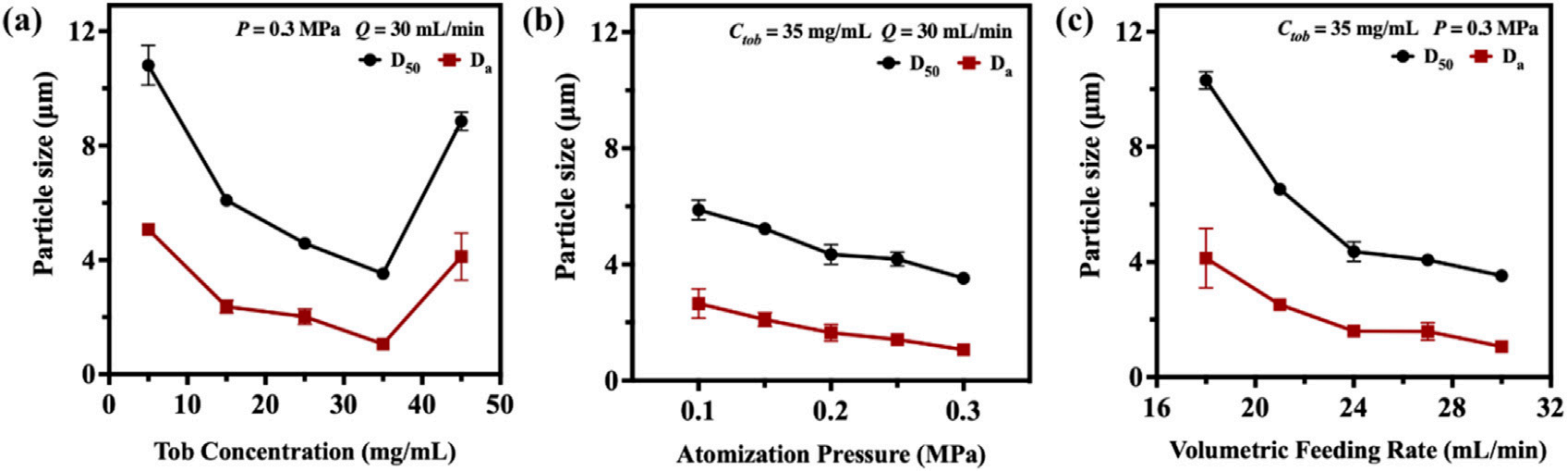
The relationship between particle size with **(a)** tobramycin concentration *C*_*tob*_; **(b)** atomization pressure *P* and **(c)** volumetric feeding rate *Q* when applying SFD method.

Generally, larger DPIs particles (>5 µm) are easily deposited in the mouth or wall of the upper respiratory tract. In contrast, smaller particles (<0.5 µm) can be expelled from the lungs during exhalation because of their smaller volume and lower inertia tract (Hebbink et al., 2022). Particles ranging from 0.5 to 2 µm are considered to be effectively deposited in deeper pulmonary (Huang, 2023). In this study, the SFD method was applied to prepare tobramycin DPIs microparticles with sizes ranging from 1 to 5 µm. The D_50_ and D_a_ values of the DPIs samples were measured using DLS and are shown in Supplementary Table S2. The DPIs prepared by the SFD method exhibited smaller particle sizes and narrower distributions than API. To investigate the effects of operation parameters, DPIs samples were prepared with different Tob concentration (Sample A1-A5; *C*_*tob*_ = 5, 15, 25, 35 and 45 mg/mL), atomization pressure (Sample A6-A10; *P* = 0.1, 0.15, 0.2, 0.25 and 0.3 MPa), and volumetric feeding rate (Sample A11-A15; *Q* = 18, 21, 24, 27 and 30 mL/min) when applying SFD method. According to Figure 1a, significant decline of D_50_ (from 10.81 ± 0.70 μm to 3.52 ± 0.04 μm) and D_a_ (from 5.07 ± 0.10 μm to 1.06 ± 0.04 μm) can be obtained when increasing *C*_*tob*_ from 5 mg/mL to 35 mg/mL. However, further increasing *C*_*tob*_ to higher value (45 mg/mL), the value of D_50_ and D_a_ improved to 8.85 ± 0.32 μm and 4.12 ± 0.82 μm, respectively.

When *C*_*tob*_ was ranging from 5 mg/mL to 35 mg/mL, an increase in the feeding solution’s viscosity was observed, which consequently led to higher surface tension. Therefore, smaller droplets and ice crystals were formed during the spray- and freeze-drying processes, which would eventually translate into smaller particles. However, when the concentration was further increased to 45 mg/mL, the particle size was primarily determined by intermolecular forces. Tobramycin is a water-soluble aminoglycoside antibiotic, and its hydroxyl and amino groups can form intermolecular hydrogen bonds during freeze-drying. This enhances the van der Waals forces between particles, leading to particle aggregation due to mutual attraction. Thus, the increased concentration contributed to the strengthening of powder interactions, resulting in larger particles (Farinha et al., 2023; Mueannoom, 2012).

Figure 1b shows that the nebulizer pressure slightly affects the DPIs particle size. The D_50_ and D_a_ particle size decreased by 40.14% (from 5.88 ± 0.34 μm to 3.52 ± 0.04 μm) and 60.00% (from 2.65 ± 0.51 μm to 1.06 ± 0.04 μm) in our experiment when the nebulizer pressure was tripled from 0.1 MPa to 0.3 MPa. Elevating the nebulizer pressure from 0.1 MPa to 0.3 MPa tended to result in a decrease of particle size. First, a heightened nebulizer pressure typically corresponded to a more vigorous atomization airflow, which led to a higher dispersion of liquid droplets and mitigation of inter-droplet interaction. Second, as the nebulizer pressure increased, the solution was sprayed into minute droplets with augmented surface area and surface energy, which resulted in the formation of finer particle (Farinha et al., 2023; Schappo et al., 2021).

The relationship between the volumetric feeding rate (*Q*) and particle size is shown in Figure 1c. A clear relationship exists in which a higher volumetric feeding rate leads to smaller D_50_ and D_a_ sizes. When the volumetric feeding rate increased from 18 mL/min to 21 mL/min, the particle size reduced from 10.31 ± 0.30 μm (D_50_) and 4.13 ± 1.03 μm (D_a_) to 6.53 ± 0.04 μm (D_50_) and 2.52 ± 0.19 μm (D_a_). Further increasing the volumetric feeding rate from 24 mL/min to 30 mL/min results in particle size reduction by 46.09% (D_50_, from 6.53 ± 0.04 μm to 3.52 ± 0.04 μm) and 57.94% (D_a_, from 2.52 ± 0.19 μm to 1.06 ± 0.04 μm), respectively. The heightened flow velocity effectively enhanced the atomization efficiency of the spray nozzle, facilitating the dispersion of liquid droplets into finer mist, which increased the surface area of the droplets. Thus, more droplets could quickly contact liquid nitrogen and lose water when dispersed. Simultaneously, the atomized droplets collided with the internal airflow, and the elevated feed flow velocity may have influenced the airflow within the system, altering the suspended time and dispersion extent of particles in the air. This reduced the aggregation of particles, which helped the droplets to form smaller particles. After preliminary screening and optimization, A4 was used in further research to evaluate their fluidity and aerodynamic performance.

**FIGURE 2:**
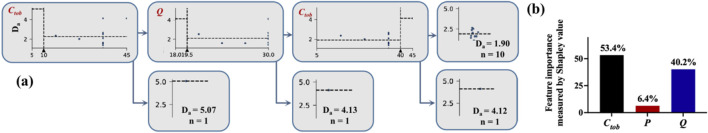
Machine learning for screening key process parameters: **(a)** schematic diagram of the decision tree model by SFD; **(b)** proportion of importance of process parameters by SFD.

To explore the connection between Tob concentration (*C*_*tob*_), atomization pressure (*P*), and volumetric feeding rate (*Q*), and pinpoint the key factor influencing the particle size of D_a_, we applied a machine learning approach, specifically a decision tree regressor, to construct a model for the research problem at hand. In this model, *C*_*tob*_, *P*, and *Q* acted as features (independent variables), whereas D_a_ served as the target (dependent variable). The model demonstrated a performance metric with a squared error of 1.23, computed as the mean across 10 simulations with fine-tuned hyperparameters. To ensure a well-rounded model and prevent overfitting, we set the minimum depth of the decision tree to 4. The resulting decision tree is visually represented in Figure 2a, where the nodes represent the experimental conditions and illustrate the distribution of samples across these conditions. Moreover, our investigation delved into the significance of each factor, as shown in Figure 2b, demonstrating that *C*_*tob*_ played a primary role, while *Q* assumed secondary importance. This observation was confirmed by decision tree analysis, where *C*_*tob*_ was identified as the root node, underscoring its pivotal role in influencing the overall outcomes. Through the evaluation of process parameters, it is demonstrated that in the subsequent more refined process optimization, the upper limit of the increased *C*_*tob*_ applicable to the SFD method is 35 mg/mL. Additionally, the *Q* is a factor worthy of further exploration. Given the flow rate limitations of the experimental equipment, it is feasible to further increase the volumetric feeding rate (>30 mL/min) by improving the equipment, so as to explore a more optimal formulation.

The pulmonary delivery and deposition efficiency of DPIs rely not only on the particle size but also on their morphology, density, fluidity, and other properties (Huang, 2023). Therefore, according to the above results and discussion, samples A4 was chosen for further evaluation of their fluidity and aerodynamic performance. The yield, density, angle of repose, and Carr index of A4 were investigated. The yield of A4 was 63.21 %± 2.11%, and the angle of repose, the Carr index value of sample A4 were 36° and 37.54%, respectively. Overall, sample A4 shows slightly good flow characteristics and powder fluidization behavior, which play a crucial role in the aerosol performance of the DPIs (Liu, 2024).

**FIGURE 3:**
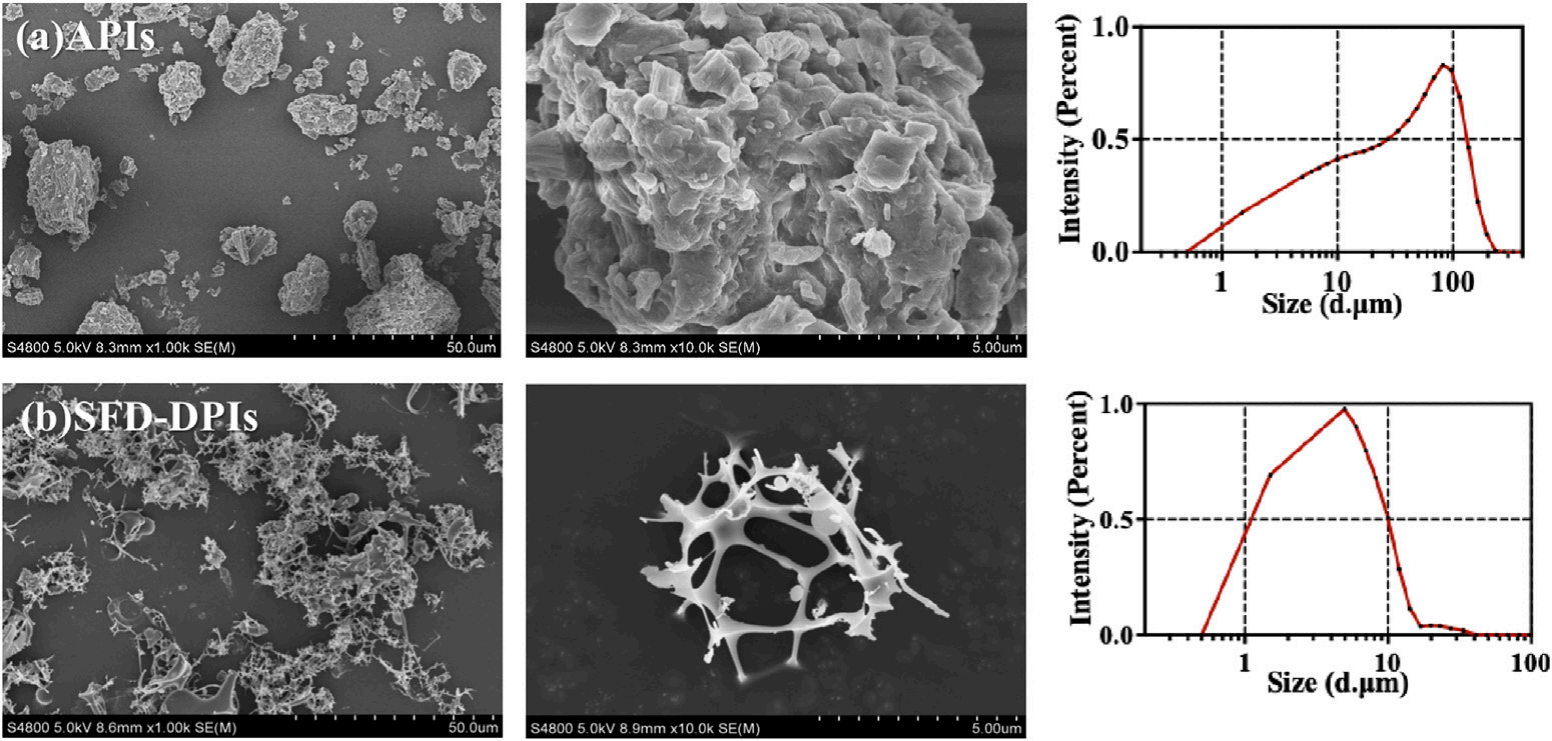
The SEM images and size distribution of **(a)** tobramycin API; **(b)** sample A4.

SEM images of the tobramycin API and samples A4 were obtained to characterize the particle size, microstructure, and morphology. As shown in Figure 3a, raw tobramycin exhibited a larger average particle size of 25.26 µm (D_50_) with an irregular shape and broader size distribution. In contrast, sample A4 showed the smallest D_50_ size (3.52 µm), narrower size distribution, and reticular spherical morphology with a loose and porous structure (Figure 3b). The formation mechanism can be explained as follows: The droplets generated in the spray process were frozen instantaneously to form spherical ice crystals after contact with liquid nitrogen. Subsequently, water was removed through ice sublimation during the freeze-drying process, resulting in the formation of a fine porous structure. Previous studies have demonstrated that this morphology is more suitable for lung drug delivery because the porous structure can improve the specific surface area of particles, leading to an increased contact area between the solvent and particles. Moreover, its low density results in a small aerodynamic diameter, which can make DPIs delivery deeper into the lungs (Ye et al., 2017).

### 3.2 Thermoanalysis and powder crystallinity

**FIGURE 4:**
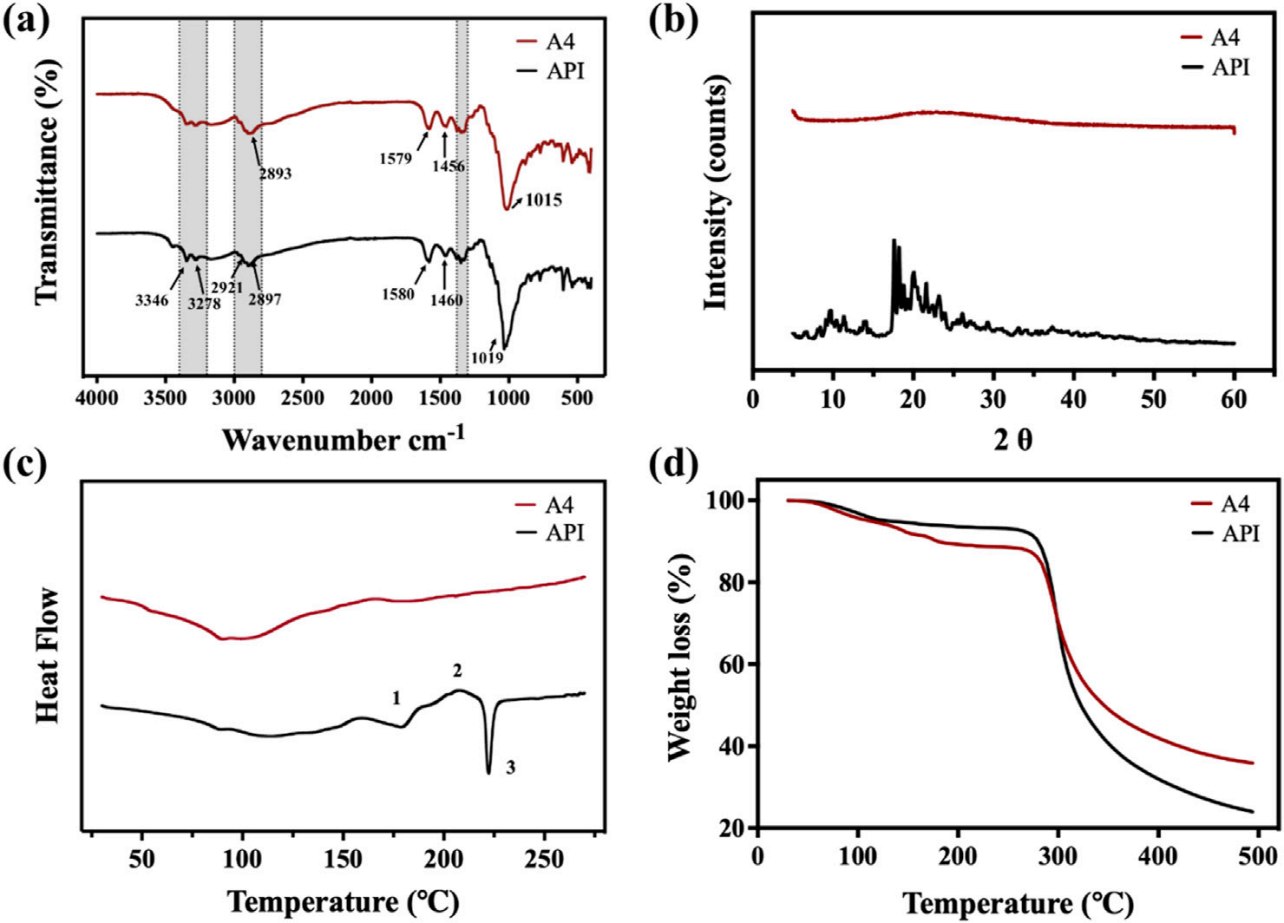
**(a)** ATR-FTIR spectrum; **(b)** XRPD spectrum; **(c)** DSC curves; **(d)** TGA curves of tobramycin API, and sample A4.

The ATR-FTIR, XRPD, DSC, and TGA results of the API and samples A4 are shown in Figure 4. The results of the ATR-FTIR spectrum (Figure 4a) showed that the absorption and intensity of the drug substance and tobramycin granule sample was consistent with the API, indicating that the SFD processes did not change the original structure. Specifically, the spectrum exhibited bands at 3,400–3,200 cm^−1^ attributable to N–H or O–H stretching, and bands at 3,000–2,800 cm^−1^ attributable to C–H stretching. The spectrum simultaneously showed a band at 1,550–1,600 cm^−1^ due to N–H bending, a typical band around 1,460 cm^−1^ caused by CH_2_ scissoring, bands at 1,300–1,380 cm^−1^ caused by O–H in-plane bending vibration, and a band around 1,019 cm^−1^ caused by C–N or C–O stretching.

The crystallinity of the API and sample A4 were analyzed using XRPD, and the results are shown in Figure 4b. It is obvious to see the perfect crystalline of tobramycin API. The XRPD pattern of tobramycin API has obvious peak intensities. In contrast, there were no peaks in the XRPD spectrum of sample A4, indicating that the SFD produced an amorphous powder, which might be caused by the rapid arrangement of tobramycin molecules in the process of SFD. Moreover, it has been demonstrated that drugs in an amorphous state generally show enhanced solubility. Thus, DPIs in an amorphous state could be released rapidly after deposition in the lung, leading to a relatively higher drug concentration and improved treatment effect (Guo et al., 2023).

The DSC curves of the Tobramycin API and samples A4 are shown in Figure 4C. Compared with previous studies (Fernández, 2018; Rosasco, 2018), the DSC curve of Tobramycin API exhibited two endothermic peaks at ∼170 and ∼230°C and one exothermic peak at ∼210°C. These three thermal events in Figure 4c are marked as 1-3, corresponding to the first endothermic peak 1, which can be ascribed to the melting of the metastable form of tobramycin, and exothermic peak 2, which was due to the stable anhydrous form of crystallization before melting. Corresponding to the melting behavior, the DSC curve showed a sharp endothermic peak three at ∼230°C. In summary, tobramycin API showed two different crystal phases at ∼170 and ∼230°C, with a recrystallization peak between the two points and degradation starting at the second melting point peak. In contrast, the thermogram of sample A4, which was prepared by the SFD method, had no endothermic peak at approximately 230°C, which is consistent with the results of similar technology and processes in other studies (Miller et al., 2015).

The TGA curves of the tobramycin API and samples A4 are shown in Figure 4d. The mass loss before 200°C can be attributed to the loss of moisture and volatile components in the particles, dehydration reactions, and release of water molecules. The deviation of the two curves was precisely the result of changes in the crystal shape. In addition, tobramycin is known to be hygroscopic in nature (Patere, 2018). As sample A4, which is in an amorphous state, is more easily soluble, the decrease in sample A4 was greater than that of API. This result was in line with expectations. However, when the temperature rose to approximately 285 °C, the curves decreased dramatically, which corresponded to the mass loss resulting from the collapse of the tobramycin structure. From the perspective of thermal stability, the remaining weights after dehydration at 285 °C were close to 90%, similar to that of tobramycin API. Therefore, samples A4 exhibited good thermal stability.

### 3.3 *In-Vitro* aerosol performance

**FIGURE 5:**
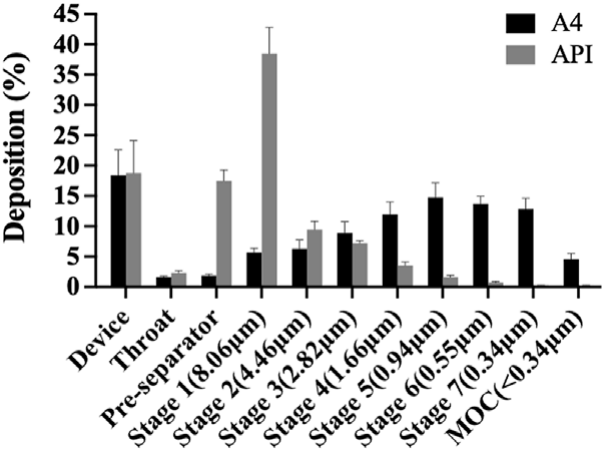
*In-vitro* aerosolisation performance of API and A4 by next-generation impactor (NGI).

The *in vitro* aerosol performance of tobramycin API and DPIs samples (A4) were tested by NGI, as shown in Figure 5. Aerosol performance and particle characteristics are listed in Table 1. The interstage distribution map shows that sample A4 was mainly deposited in stages 4–7, making these particles very suitable for delivering the drug to the lungs. The EF range of API was 97.91%, which was much higher than that of A4 (89.02%). This difference can be attributed to the fact that the particles prepared by the SFD method were loose and porous. Meanwhile, the phenomenon of electrostatic adsorption can be observed during the deposition experiment *in vitro* (Kaialy, 2016). It might stem from the fact that the edges or tips of the pores in porous particles are prone to form charge accumulation, leading to an increase in the local charge density.

**TABLE 1:** Aerodynamic performance parameters of API and A4.

	EF (%)	FPF (%)	MMAD (μm)	GSD	D_50_ (μm)	D_a_ (μm)
API	97.91 ± 0.77	18.65 ± 1.04	12.51 ± 1.45	3.90 ± 0.94	25.26 ± 3.29	27.31 ± 1.60
A4	18.65 ± 1.04	83.31 ± 3.97	1.30 ± 0.15	3.42 ± 0.62	3.52 ± 0.04	1.06 ± 0.04

Although API have excellent fluidity, and they are easier to inhale and deposit *in vivo* and *in vitro*, which is equivalent to a lower dose loss when inhaled tobramycin DPIs (Sun et al., 2020). On the other hand, the FPF of samples A4 was 83.31%, which were significantly higher than those of the APIs (18.65%). Meanwhile, the MMAD of samples A4 was1.31 μm, which is much higher than the APIs valued 12.51 μm, indicating that the size of inhalable particles is best transported to the respiratory bronchioles and alveoli with small deviations through sedimentation and diffusion (a target for the treatment of *Pseudomonas aeruginosa* infection CF by inhaling tobramycin). In addition, the GSD value indicates the polydispersity of the particle-size distribution. The GSD value of A4 was lower than API, implying that it was suitable for pulmonary administration (Huang, 2023). Therefore, this study paves the way for the effective treatment of excipient-free tobramycin DPIs through the lung pathway in future CF.

**FIGURE 6:**
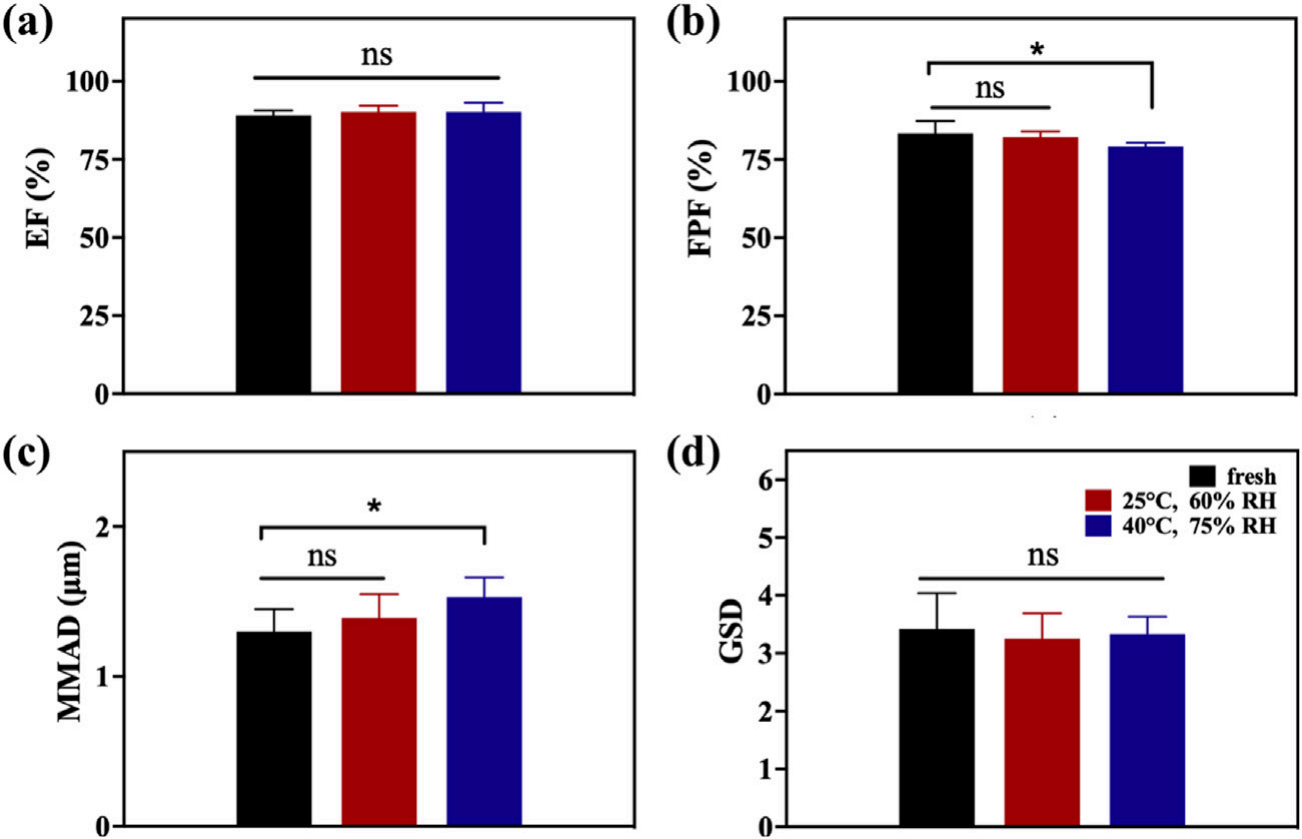
EF **(a)**, FPF **(b)**, MMAD **(c)**, and GSD **(d)** of A4 after stability testing after storage under different conditions for 28 days.

To determine the influence of storage stability, A4 was stored in commercial packaging boxes which were airtight and light-proof for 28 days under two conditions: 25°C and 60% relative humidity (RH) and 40°C and 75% RH. After testing the storage stability, the aerodynamic performance parameters of all samples were measured, as shown in Table 2 and Figure 6, and compared with the values of fresh DPIs samples to evaluate the stability of particle storage. As the temperature and humidity of the storage environment increased, the EF range of the stored samples slightly increased owing to the increase in density after moisture absorption, overcoming electrostatic adsorption and reducing particle gaps, resulting in a slight improvement in flowability, although there was no significant difference under various conditions. Moreover, because amorphous powder is more hygroscopic than crystalline powder, increasing the humidity results in particle agglomeration after the absorption of water molecules, and the higher the humidity, the more obvious the agglomeration phenomenon. Previous studies have reported that crystallization/recrystallization of an amorphous form is a complex phenomenon governed by many factors, including intermolecular interactions such as hydrogen bonding and/or π–π interactions, and ionic interactions between two molecules can form in some amorphous particles (Lu, 2019; Müller, 2015). A higher water content may lead to particle growth owing to an increase in interparticular forces (e.g., capillary forces). This led to a significant decrease in FPF (p < 0.05) and a significant increase (p < 0.05) in MMAD of samples stored at 40°C and 75% RH for 28 days compared with the fresh sample. Based on the 28-days storage stability results, A4 shows relatively superior results. However, in order to ensure the long-term stability of A4, it is recommended to use materials with excellent moisture-proof performance for the primary packaging to prevent moisture absorption. In addition, desiccant packets can be considered for addition in the secondary packaging to further control the internal humidity environment.

**TABLE 2:** Effect of storage conditions on aerodynamic performance.

	EF (%)	FPF (%)	MMAD (μm)	GSD
Fresh	89.02 ± 1.59	83.31 ± 3.97	1.30 ± 0.15	3.42 ± 0.62
25°C, 60% RH	90.18 ± 1.99	82.18 ± 1.74	1.39 ± 0.16	3.25 ± 0.44
40°C, 75% RH	90.13 ± 3.00	79.16 ± 1.12	1.53 ± 0.13	3.33 ± 0.30

### 3.4 *In-vitro* and in-vivo safety and activity

#### 3.4.1 MTT-cytotoxicity assay

**FIGURE 7:**
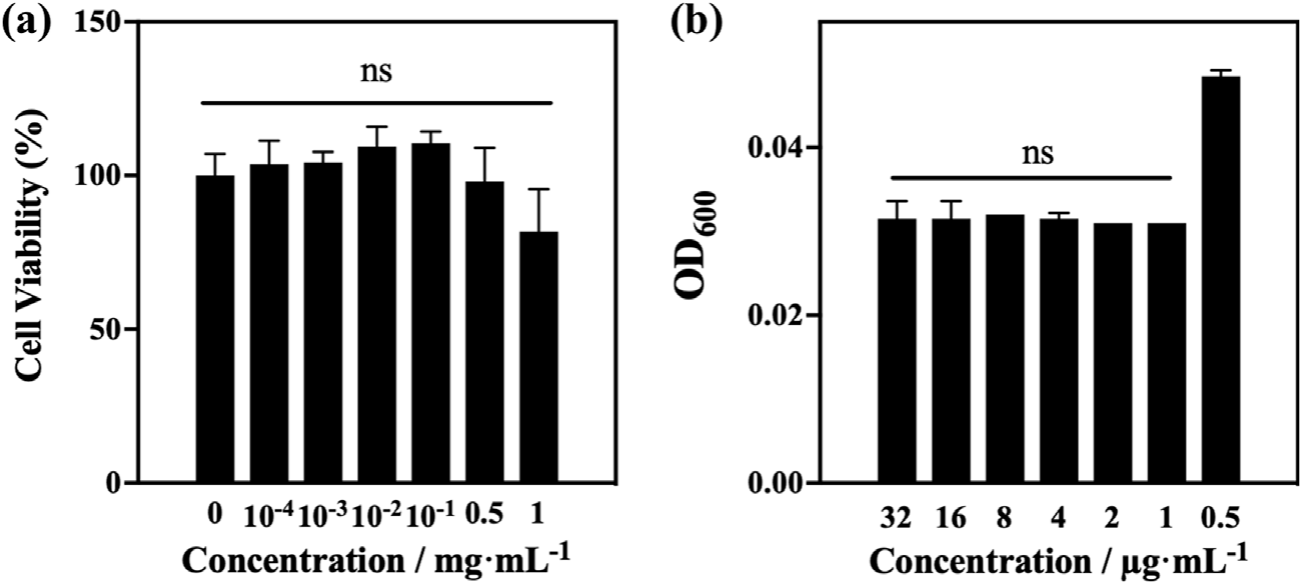
**(a)** MTT test results of optimum samples A4; **(b)** *in vitro* bacteriostatic test results of optimum samples A4.

To represent the environmental characteristics of the peripheral lung epithelium at the expected delivery area of tobramycin DPIs (Mukhtar et al., 2021), the A549 cell line was selected for *in vitro* safety evaluation (Figure 7a). A549 human alveolar cells were incubated and exposed to samples A4 at different concentrations (1, 0.5, 10^−1^, 10^−2^, 10^−3^, and 10^−4^ mg/mL). Overall, the inhibitory cell concentration (IC) of A4 was more than 50%. Cell viability values in the range of 10^−4^ to 0.5 mg/mL were more than 90%, and cell growth was not significantly inhibited. Generally, substances with an *in vitro* IC50 exceeding or equal to 1 mg/mL in A549 cells are unlikely to be toxic during animal or human research on drug/formulation development (Adhikari et al., 2022; Anderson et al., 2013). Therefore, it can be concluded that samples A4 has good tolerance to A549 cells, indicating that they are non-toxic. This indicates the safety and feasibility of using tobramycin DPIs in further research.

#### 3.4.2 Evaluation of antibacterial activity

*Pseudomonas aeruginosa* is one of the most common pathogens that cause pulmonary infections in patients with cystic fibrosis (Jennings et al., 2021; La Rosa et al., 2021). Therefore, the antibacterial activities of A4 was tested against *P. aeruginosa* with a streptomycin-resistant bioluminescent plasmid. After co-incubating with diluted bacterial solution for 24 h, the OD_600_ values of A4 showed significant changes at a concentration of 0.5 μg/mL, indicating that tobramycin products exhibited significant activity against *P. aeruginosa* strains with a MIC of 0.5 μg/mL (Figure 7b).

#### 3.4.3 *In-vivo* safety study

**FIGURE 8:**
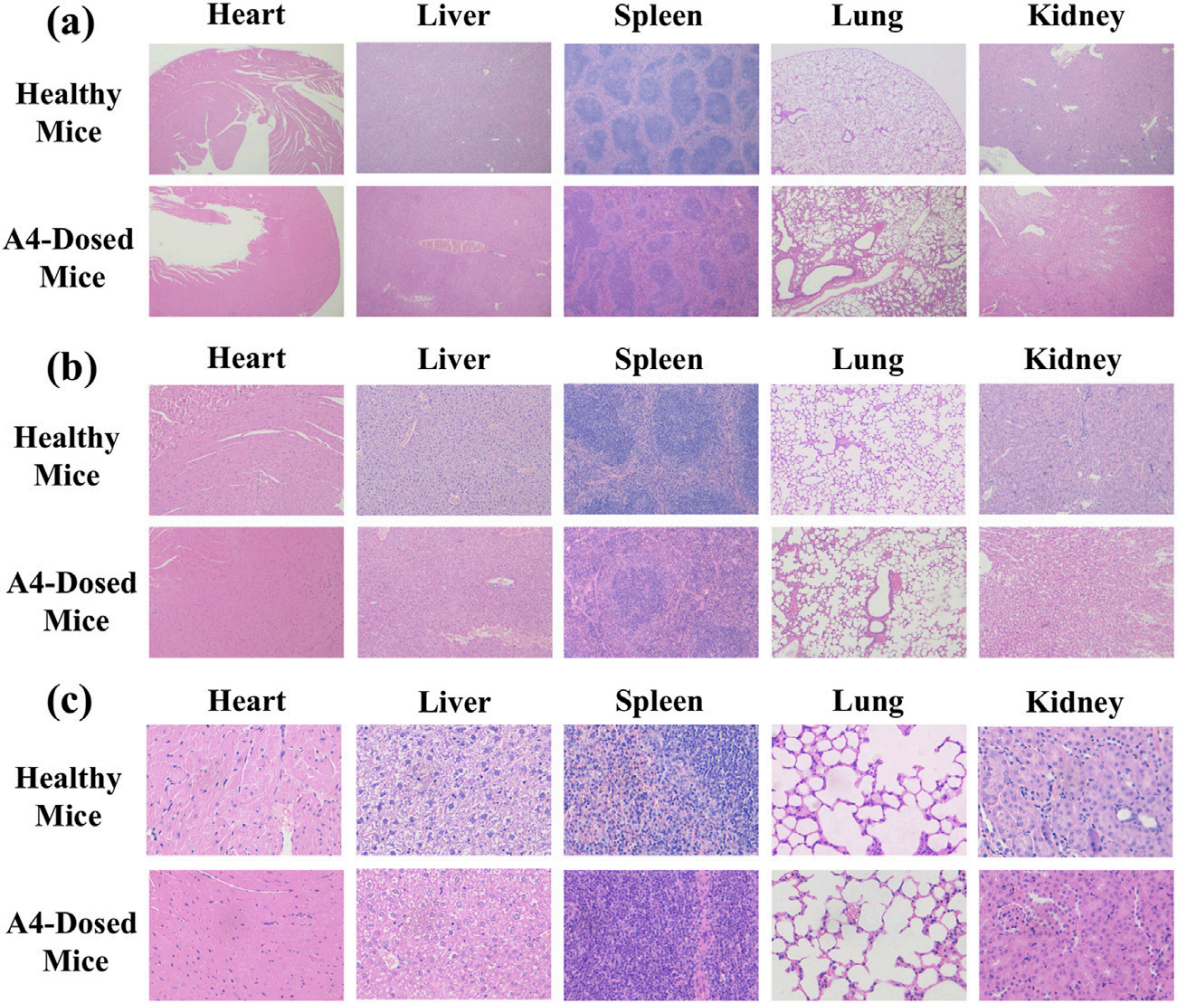
*In-vivo* safety analysis. 4 x **(a)**, 10 x **(b)**, 40 x **(c)** Morphological images of the main organs of healthy and dosed mice.

To ensure that the dosage used in the subsequent efficacy evaluation was within the safe range, a 14-day inhaled toxicology study was conducted. No mice died within 14 days after delivering A4 in high-dose lungs (30 mg/kg) into the trachea every day, indicating that tobramycin DPIs had no acute toxicity to mice. Meanwhile, there was no significant difference in body weight and organ weight between healthy mice and mice that inhaled high-dose tobramycin A4 (Table 3). Autopsy of the internal organs showed no macroscopic differences in size, color, or texture between healthy mice and mice in the high-dose tobramycin A4. Moreover, no pathological signs or gross lesions were observed in important organs. In addition, the as-synthesized DPIs did not cause any treatment-related adverse reactions in the mice during the treatment period. Histological examination also showed no relevant changes in the heart, liver, spleen, lungs, and kidneys in all groups (Figure 8). In addition, there were no significant adverse effects on food consumption and behavior between the group of mice treated with high-dose tobramycin DPIs.

**TABLE 3:** The body weight and organs weight of healthy mice and dosed mice (Data presented as mean ± SD, n = 6).

	Healthy mice	A4-dosed mice
Body weight (g)	27.61 ± 0.51	27.26 ± 0.36
Heart weight (g)	0.1565 ± 0.0032	0.1529 ± 0.0036
Liver weight (g)	1.2124 ± 0.0130	1.2090 ± 0.0165
Spleen Weight (g)	0.0852 ± 0.0031	0.0847 ± 0.0031
Lung Weight (g)	0.1801 ± 0.0034	0.1761 ± 0.0036
Kidney Weight (g)	0.3239 ± 0.0129	0.3100 ± 0.0071

### 3.5 *In-vivo* pharmacodynamics studies

#### 3.5.1 Survival rate and behavioral state

**FIGURE 9:**
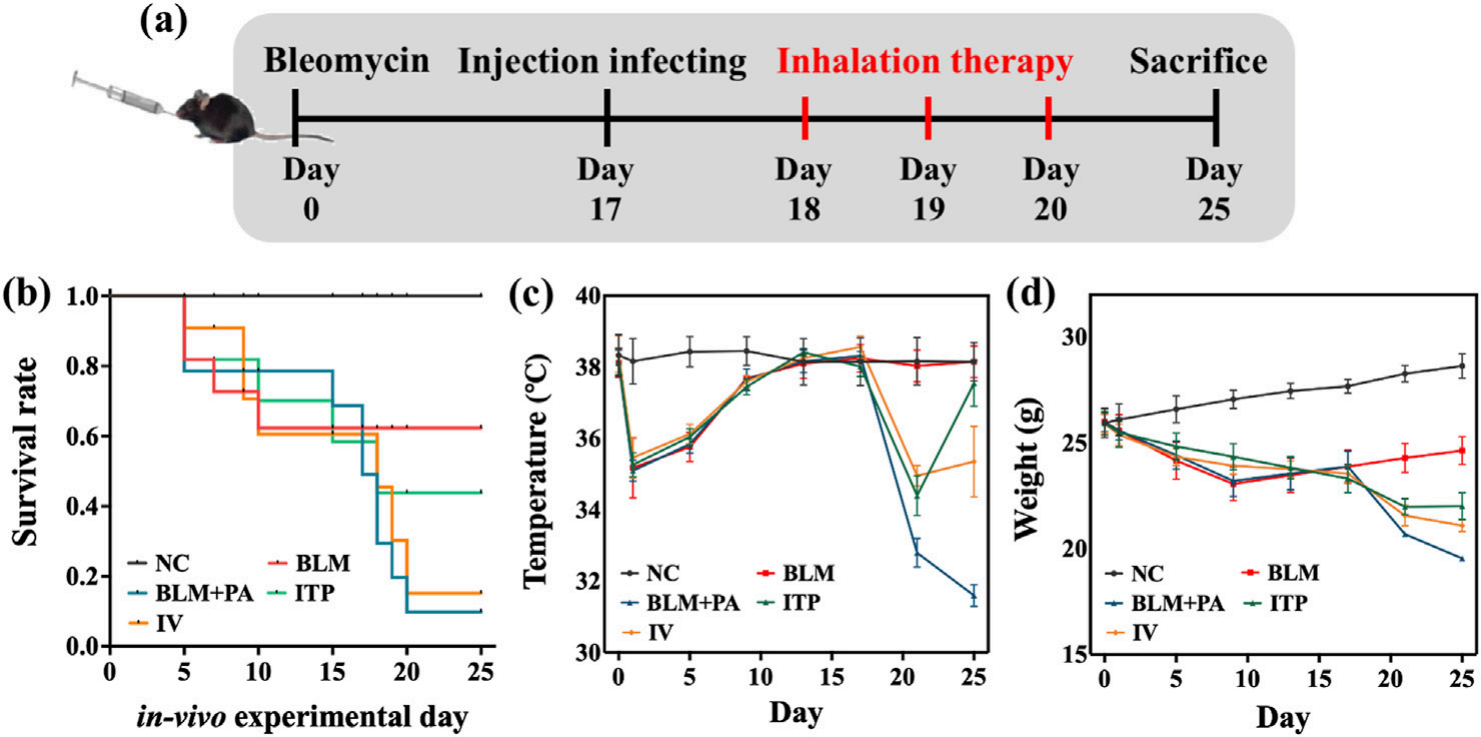
*In vivo* anti-infection analysis: **(a)** Flow chart of the animal experiment; **(b)** Kaplan-Meier chart; **(c)** weight; and **(d)** body temperature during the animal experiment.

We induced disease mouse models that had cystic fibrosis with pulmonary *Pseudomonas aeruginosa* infection (Figure 9a) to investigate the *in vivo* pharmacodynamics of the inhaled tobramycin DPIs prepared in this study. Figure 9b shows the Kaplan–Meier plot after 25 days of injection and treatment. No deaths were observed in negative control (NC) mice. At the end of the study period, the survival rates in the BLM, BLM + PA, ITP, and IV groups were 0.67, 0.17, 0.58,and 0.42, respectively. The graph shows that intratracheal DPIs administration and intravenous injection of tobramycin improved the survival rate of mice.

Both the weight (Figure 9c) and body temperature (Figure 9d) of the NC group remained stable during the efficacy validation period. After 1 day, the body temperature and weight of the BLM, BLM + PA, ITP, and IV groups dropped, and then slowly ascended back. After *P. aeruginosa* infection on day 17, the body temperature and weight of the BLM + PA, ITP, and IV groups dropped steeply. Decreased body temperature in animals is often an important sign of increased disease severity. When the core body temperature drops by 4°C or more, this may indicate that the animal has worsened after infection with pathogens. As shown in Figure 9d, the temperature of the uninfected mice remained almost unchanged, and the temperature of the infected mice decreased by more than 4 °C, indicating to a large extent that the mice were severely infected (Ren et al., 2022). Most untreated mice with body temperatures below 32 °C died from infection. Additionally, infected mice showed signs of distress, such as curling up, lethargy, slow movement, loss of appetite, or other unhealthy behaviors, indicating that the disease condition deteriorated after being attacked by infected pathogens. The average body temperature of the all-treated group increased after treatment, and even that of the ITP group was close to that of the BLM group at the end of the study, which indicated that the DPIs treatment by sample A4 had attained a significant effect.

Furthermore, it is known from the results that under the condition of an equal dose, the DPIs sample prepared by SFD has a better therapeutic effect. Based on the Noyes-Whitney and Freundlich-Ostwald equations, reducing the particle size can increase the specific surface area and significantly improve the saturation solubility and dissolution rate (Wang, 2023). The better efficacy of sample A4 is likely due to its hollow reticular structure, which is more suitable for inhalation at the lesion site. Simultaneously, because of its amorphous state and higher solubility, sample A4 can quickly increase the local concentration in a short time to achieve a better therapeutic effect.

#### 3.5.2 *In-vivo* imaging of the infection

**FIGURE 10:**
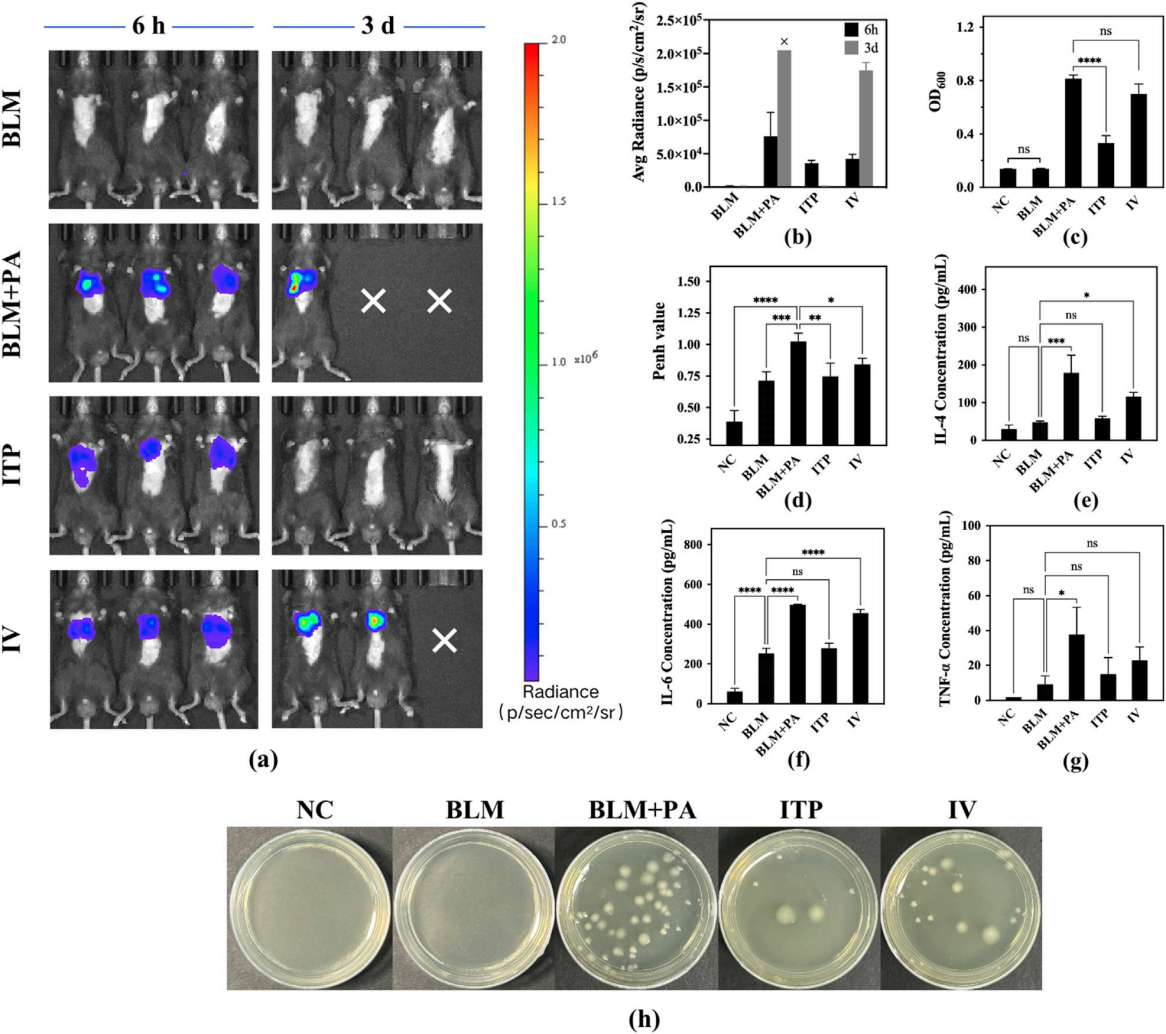
**(a)** Bioluminescence images in the mice at 6h and 72h (“×” means the experiment animals died because of infection.) **(b)** Total radiance count of bioluminescence images in mice of different groups; **(c)** OD_600_ of bacterial solution from different groups; **(d)** Penh value in mice of each group after drug administration. Determination of inflammatory factors in mouse alveolar lavage fluid from different groups: **(e)** IL-4, **(f)** IL-6, and **(g)** TNF-α. **(h)** Photographs of bacterial colonies of lung homogenates from the *P. aeruginosa*-infected lung tissues of different groups. (Level of significance: * Indicating the significant difference with p < 0.05, ** Indicating the significant difference with p < 0.01, *** Indicating the significant difference with p < 0.001, **** Indicating the significant difference with p < 0.0001).

A mouse disease model was established by infecting lung tissues with *P. aeruginosa*. The inhibitory efficacy of samples A4 against infection was evaluated by inhalation or intravenous injection. After inhalation of A4 (ITP group) in mice, the accumulation of *P. aeruginosa* in the lungs was significantly reduced, the deterioration of the disease condition was delayed, and the survival rate of mice with pneumonia was improved simultaneously (Figure 10a). From the perspective of quantified *in vivo* biological luminescence indicators, the infection of the ITP group of mice completely disappeared, and the levels were consistent with those in the BLM group. In contrast to the BLM group, intravenous injection also played a role in alleviating the disease and reducing infection, but the disease situation in mice was still very serious. The average radiance was 1.7 × 10^5^ (p/s/cm^2^/sr), while the values for the ITP group was 1.5 × 10^3^ (p/s/cm2/sr) on day 3 (Figure 10b). In conclusion, inhaled administration showed a higher anti-*P. aeruginosa* efficacy than intravenous injection.

#### 3.5.3 Enumeration of bacterial load of lungs

*Pseudomonas aeruginosa* was successfully cultured from the lungs of infected mice, as only high CFU can be produced from lung tissue homogenates to estimate the bacterial burden. The results shown in Figures 10c,h matched well with the mouse survival rate (Figure 9b), body weight (Figure 9c), and body temperature (Figure 9d). Owing to the lack of treatment in the BLM + PA group, the lung bacterial count was the highest. BLM group was not inoculated with *Pseudomonas aeruginosa*; therefore, there was no bacterial growth. The OD_600_ value was consistent with that of the NC group, and there were significant differences between the ITP group value of 0.333 and BLM + PA group value of 0.815 (p < 0.0001). Among them, the best treatment effect was shown in the ITP group which is higher than IV group, but it did not recover to the same level as the NC and BLM groups. Therefore, to completely eradicate the infection, it is necessary to consider strengthening the dosage, increasing the frequency of administration, or extending the treatment time to enhance efficacy.

#### 3.5.4 Enzyme-linked immunosorbent assay

Cytokines, including IL-4 (Figure 10e), IL-6 (Figure 10f), and TNF-α (Figure 10g), were quantified using an ELISA kit. IL-4, IL-6, and TNF-α Cytokines are involved in pulmonary fibrosis following *P. aeruginosa* infection (Cao et al., 2022; Wu et al., 2021). Compared to the BLM + PA group, the levels of all cytokines improved after treatment, and the values of the ITP group were similar to those of the BLM group, indicating that the efficacy of inhaled amorphous powder can reduce the levels of inflammatory factors in mice before infection. ITP group had significantly lower values than the IV group. On this basis, it was confirmed that pulmonary administration was superior to intravenous administration.

#### 3.5.5 Morphometric analysis of lung

**FIGURE 11:**
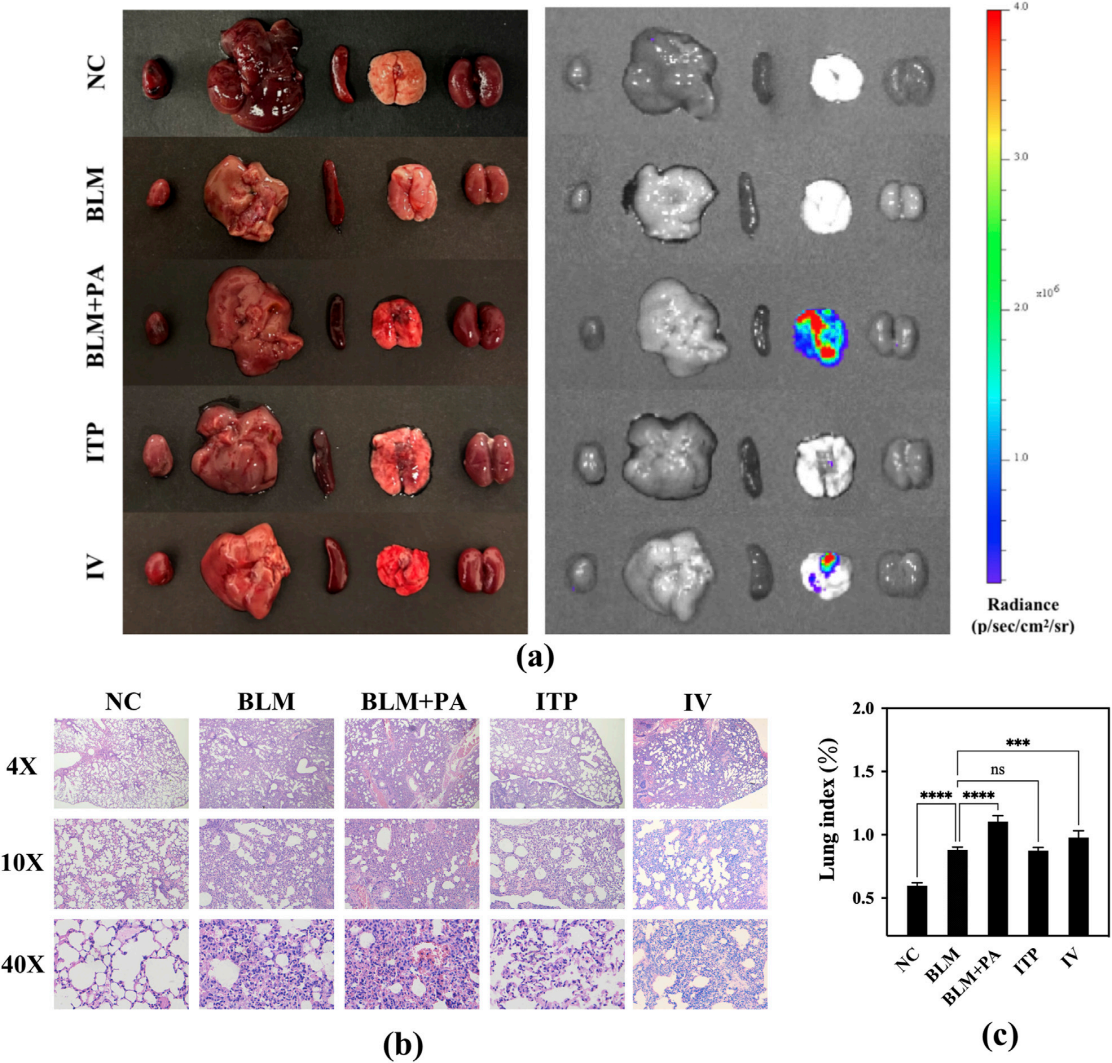
**(a)** Bioluminescence images of the main organs of mice; **(b)** morphological changes in the lungs of mice after dosing; **(c)** lung index of mice.

The lungs of the BLM + PA group imaged using the *In Vivo* Imaging System (IVIS) showed higher bioluminescence intensity, and there were also severe phenomena of edema and congestion from a macro perspective (Figure 11a). Figure 11b shows the histological evaluation results of the collected lungs, which confirmed the severity of fibrosis in the BLM group. The BLM + PA group showed severe lung injury without any normal alveoli or septa, which were replaced by fibrous masses with no observable gaps. In contrast, the ITP group showed a healthier lung morphology, and the appearance of its alveoli was maintained, indicating a significant improvement in the disease condition. Additionally, the disease situation including septal collapse, alveolar merger, and increased septal thickness in damaged and infected lungs were improved slightly in IV group compared to the BLM + PA group, but there was still a large amount of diffuse exudate of red blood cells in the lungs. Compared to the intravenous injection group, the ITP group treated through the lung route showed a morphology closer to the normal alveoli of the NC group. Meanwhile, the lung image showed a bioluminescence intensity that was close to that of the NC and BLM groups. These results confirmed that inhaled tobramycin DPIs were more effective than the tobramycin solution used for intravenous administration.

The lung index can dynamically reflect the progression of lung disease. With the development of lung inflammation and fibrosis, the exudation and water content of the lungs increases, various proteins infiltrate the lung tissue, and the collagen content of the lungs increases, leading to an increase in lung weight, so the lung index also rises accordingly. The lung index data for the different groups of mice are shown in Figure 11c. Compared to the NC group’s 0.5965, BLM group developed pulmonary fibrosis in their lungs, resulting in a significantly higher lung index value of 0.8813 (p < 0.0001) due to pulmonary edema. Based on pulmonary fibrosis, *P. aeruginosa* was introduced into the lungs, causing severe infection in the BLM + PA group mice, resulting in more obvious inflammation and edema. The lung index of this group (1.1042) was also markedly higher than that of the BLM group (p < 0.0001). All treatment groups showed efficacy, with the inhalation groups showing better efficacy than the intravenous injection group. The lung index levels in the ITP group (0.8744) decreased to very close to those in the BLM group.

#### 3.5.6 Whole body plethysmography test penh value

Figure 10d shows the results of the evaluation of the improvement in airway resistance in mice using WBP. In the BLM + PA group, air circulation and oxygen exchange were difficult due to lung fibrosis and bacterial infection, leading to higher Penh values. However, the Penh value significantly decreased in the ITP group value of 0.747 (p < 0.01), and IV group value of 0.842 (p < 0.05) compared to that in the BLM + PA group value of 1.025. Specifically, the Penh values were more significantly decreased in the ITP group, in which tobramycin at the same dose was administered through the lung delivery route, than in the IV group. The improvement in the Penh value proved the effectiveness of tobramycin administration (Kang et al., 2022), and a better improvement effect was observed in the group receiving tobramycin treatment via the pulmonary route. These results are similar to those of previous studies on mouse status, infection status, and pathological characteristics, which may be because of the effective concentration of tobramycin reaching the lungs and demonstrating substantial drug efficacy.

Based on the *in vivo* evaluation, it was evident that inhalation administration also exhibits better therapeutic effects. From the perspective of DPIs and powder science, the advantages of sample A4 in terms of microstructure and physical and chemical properties are analyzed and summarized as follows: DPI particles prepared by the spray freeze-drying method have extremely low density, which can cause the particles to obtain a lower aerodynamic particle size with extremely low density under the same conditions, thus improving delivery efficiency in the lungs. At the same time, DPI particles prepared by the spray freeze-drying method have extremely strong re-solubility, because their porous structure gives the particles a very high specific surface area, which is instrumental in increasing the contact area of the solvent. Additionally, the porous surface structure of the DPIs makes themselves have excellent aerodynamic performance similar to “golf balls”, so they can be transported faster and farther under the same transmission power (Moriyama, 2023). Moreover, inhalation administration exhibits better therapeutic effects than intravenous administration, possibly due to the compatibility of the lung structure with inhalation administration. On the one hand, from the perspective of lung morphology and structure, the number of pulmonary alveoli in the lungs is numerous, resulting in a high specific surface area. In addition, the lungs have abundant capillaries, making them a physiological structure with a high blood flow. Furthermore, inhalation administration, which is a non-invasive method of drug delivery, can directly deliver drugs to the lungs. Compared with traditional drug delivery methods, such as intravenous administration, it reduces the systemic distribution of the drug, accelerates the increase in drug concentration in the lungs, and allows the drug to be quickly absorbed through the lungs and enter the systemic blood circulation, resulting in greatly improving the distribution of the drug in the body. Furthermore, DPIs in this study demonstrated extremely excellent therapeutic effects. Especially when compared with some clinical and scientific research, the DPI administration method used in this study is significantly superior to liquid nebulization inhalation in terms of dosage. It can achieve therapeutic effects at a much lower dosage (Guan et al., 2023; Ramaa et al., 2021). Consequently, in the foreseeable future, efforts will be concentrated on optimizing the manufacturing processes of DPIs. This includes exploring novel materials with enhanced stability and improved aerosolization properties to further refine the particle characteristics. Additionally, more extensive *in-vivo* studies across diverse patient populations will be carried out to precisely determine the optimal dosing regimens for different disease severities and patient profiles, thus maximizing the potential of DPIs in clinical applications.

## 4 Conclusion

In this study, we developed and optimized manufacturing methods for excipient-free tobramycin DPIs. The DPIs prepared by SFD exhibited reticular spherical particles with loose and porous structures. The DPIs sample exhibited excellent safety and pulmonary delivery/deposition performances. The results of the *in vivo* study demonstrated that pulmonary delivery of tobramycin DPIs could significantly reduce the number of bacteria in the lung, alleviate inflammation-related infections, and reveal better treatment effects than intravenous injection on bacterial pneumonia. In summary, the manufacturing process and evaluation methods developed in this study can not only be used for the production of excipient-free tobramycin dry powder inhalation, providing a promising and effective strategy for it, but also be applied to the preparation techniques of other excipient-free inhaled antibiotics. This will facilitate the development of treatment methods for severe bacterial infections through pulmonary delivery, while also enabling more comprehensive and accurate assessment of the performance and quality of these products.

## Data Availability

The original contributions presented in the study are included in the article/Supplementary Material, further inquiries can be directed to the corresponding authors.
