# Supplementary material for: Pulmonary delivery of excipient-free tobramycin DPIs for the treatment of Pseudomonas aeruginosa lung infection with CF
Source: Front Pharmacol. 2025 Jun 17;16:1528905. doi: 10.3389/fphar.2025.1528905 (PMC12208834; doi:10.3389/fphar.2025.1528905)
Supplement: Supplementary file 1 [file DataSheet1.pdf]

Supplementary Material

Supplementary Table S1. Composition and conditions used to prepare the different SFD samples of tobramycin DPIs.

No.	Tob Concentration (C_{tob}, mg/mL)	Nebulizer Pressure (P, MPa)	Volumetric Feeding Rate (Q, mL/min)
A1	5	0.3	30
A2	15	0.3	30
A3	25	0.3	30
A4	35	0.3	30
A5	45	0.3	30
A6	35	0.1	30
A7	35	0.15	30
A8	35	0.2	30
A9	35	0.25	30
A10 (same as A4)	35	0.3	30
A11	35	0.3	18
A12	35	0.3	21
A13	35	0.3	24
A14	35	0.3	27
A15 (same as A4)	35	0.3	30

Supplementary Table S2. Size characteristics of samples by SFD

No.	Tob Concentration (C_{tob}, mg/mL)	Nebulizer Pressure (P, MPa)	Volumetric Feeding Rate (Q, mL/min)	D_{50} ± sd (μm)	D_a ± sd (μm)
A1	5	0.3	30	10.81 ± 0.70	5.07 ± 0.10
A2	15	0.3	30	6.09 ± 0.07	2.37 ± 0.23
A3	25	0.3	30	4.58 ± 0.20	2.02 ± 0.27
A4	35	0.3	30	3.52 ± 0.04	1.06 ± 0.04
A5	45	0.3	30	8.85 ± 0.32	4.12 ± 0.82
A6	35	0.1	30	5.88 ± 0.34	2.65 ± 0.51
A7	35	0.15	30	5.23 ± 0.18	2.10 ± 0.24
A8	35	0.2	30	4.35 ± 0.34	1.65 ± 0.29
A9	35	0.25	30	4.18 ± 0.24	1.41 ± 0.13
A10 (same as A4)	35	0.3	30	3.52 ± 0.04	1.06 ± 0.04
A11	35	0.3	18	10.31 ± 0.30	4.13 ± 1.03
A12	35	0.3	21	6.53 ± 0.04	2.52 ± 0.19
A13	35	0.3	24	4.36 ± 0.34	1.60 ± 0.13
A14	35	0.3	27	4.07 ± 0.12	1.59 ± 0.31
A15 (same as A4)	35	0.3	30	3.52 ± 0.04	1.06 ± 0.04
